# Disaster Governance for Community Resilience in Coastal Towns: Chilean Case Studies

**DOI:** 10.3390/ijerph14091063

**Published:** 2017-09-14

**Authors:** Paula Villagra, Carolina Quintana

**Affiliations:** 1 Instituto de Ciencias Ambientales y Evolutivas, Facultad de Ciencias, Universidad Austral de Chile, Valdivia 509000, Chile; 2 Laboratorio de Paisaje y Resiliencia Urbana, Universidad Austral de Chile, Valdivia 509000, Chile; contacto@pru-lab.cl

**Keywords:** disaster governance, community resilience, tsunami

## Abstract

This study aimed to further our understanding of a characteristic of Community Resilience known as Disaster Governance. Three attributes of Disaster Governance—redundancy, diversity, and overlap—were studied in four coastal towns in southern Chile that are at risk of tsunamis. Overall, we explored how different spatial structures of human settlements influence Disaster Governance. Using the Projective Mapping Technique, the distribution of emergency institutions (N = 32) and uses given to specific sites (e.g., for refuge, sanitary purposes and medical attention) were mapped. Content and GIS analyses (Directional Distribution and Kernel Density Index) were used to explore the dispersion and concentration of institutions and uses in each town. Disaster Governance was found to be highly influenced by decisions taken during regional, urban, and emergency planning. Governance is better in towns of higher order in the communal hierarchical structure. Most of the emergency institutions were found to be located in central and urban areas, which, in turn, assures more redundancy, overlap, and diversity in governance in the event of a tsunami. Lack of flexibility of emergency plans also limits governance in rural and indigenous areas. While the spatial relationships found in this study indicate that urban sectors have better Disaster Governance than rural and indigenous sectors, the influence of resource availability after tsunamis, the role and responsibility of different levels of governments, and the politics of disaster also play an important role in Disaster Governance for determining Community Resilience. These findings shed light on emergency planning and aspects of the Disaster Management cycle.

## 1. Introduction

Community Resilience refers to the capacity of systems to cope and adapt after extreme events in such a way that they maintain their basic structure, function, and identity [1,2]. Resilience also refers to a set of processes that link and guide networked capacities to assure adaptation after disturbance [3]. According to Cutter [1] the measurement and identification of standards and metrics to measure Community Resilience is a challenge for governments at all levels. This is further complicated because Community Resilience is determined by the inherent characteristics of the system and the resources it possesses. Despite this, Community Resilience “…is becoming [the] de facto framework for enhancing community level disaster preparedness, response and recovery…” [4] (p. 65). Clearly, resilience in the human environment refers to how communities efficiently prepare, plan, and adapt in the case of extreme events; hence, achieving Community Resilience implies knowing the characteristics of the territory that affect resilience, before, during and after a disturbance and taking the necessary actions to improve resilience before another catastrophic event.

One characteristic that influences Community Resilience is Disaster Governance. Effective Disaster Governance produces resilience [5]. Tierney [6] (p. 344) explains that Disaster Governance consists of “the interrelated set of norms, organizational and institutional actor(s), and practices (…) that are designed to reduce the impacts and losses associated with disasters”. Although governance has been traditionally fragmented, nowadays it encourages collective actions among all stakeholders with interest in the matter of concern. Since hazards and disasters are complex events that affect communities at all dimensions (e.g., physical, social, economic, etc.), extensive cross-national and cross-institutional collaborations are required and should be incorporated in efficient Disaster Governance [6]. Indeed, a content and cluster analysis of 1069 peer-reviewed journals [7] indicates that the characteristics that influence governance are: stakeholders’ involvement and commitment; cooperation and collaboration at a variety of scales (e.g., distribution of government functions); and flexibility, which, for example, allows for the adjustment of policies and regulations among others in case of an emergency.

Disaster Governance is also political. There is extensive literature exploring the impact of government actions on mortality rate after earthquake events [8,9,10,11,12,13]. A democratic state has been found to mitigate the effects of disasters on populations by, for example, decreasing disaster mortality rates [8,9]. Expected mortality rates decrease more rapidly when democracies are older and more established and when there is a higher per capita income [9]. Despite this, these finding may not hold for corrupt governments where, for example, a corrupt public sector can lead to the construction of buildings that collapse in major earthquakes [10]. Additionally, inequality in per capita income can also influence the number of fatalities resulting from earthquakes [11]. Inequalities can hamper the collective actions that can ensure the creation and enforcement of building codes, the retrofit of built structures, and the definition of quake-sensitive zones. These three aspects together can considerably reduce fatalities. Besides, a number of studies highlight the influence of government responsiveness in the reduction of mortality resulting from earthquakes. Indeed, countries with higher newspaper circulation and greater electoral accountability have been found to be more effective in calamity relief [13]. From this, it is seen that informed citizens can influence governments’ responses, and in these types of communities citizen preference is reflected in disaster policies. Overall, well-equipped government institutions can lower fatalities from disasters by providing effective regulations and planning and by improving the quality of infrastructure and emergency areas [12].

These studies indicate that Disaster Governance is particularly important in developing countries [14] because it can particularly affect the needs of poor communities. Aspects such as disparities in income, well-being, access to services, and political empowerment [6], as well as lack of resources, poor diversity of stakeholders, and missing links among established networks [7] can hamper the effectiveness of Disaster Governance. These disadvantages, in turn, encourage responses to disasters to focus on coordination rather than on risk reduction [15]. These shortcomings reduce the effectiveness of institutions and deteriorate the capacity to coordinate [15]. Particularly for developing countries, better Disaster Governance has been found when emergency institutions and the civil society interact. The Cuban response to Hurricane Sandy in 2012 [16] is an example of such coordination between the local community and government and non-government institutions, which resulted in a high degree of Community Resilience. The Civil Defense’s ability to act together with the inhabitants of the city and the rest of Cuba prevented greater loss of human lives. This example also sheds light on the importance of integrating extreme weather and disaster scenarios into regional planning in order to facilitate governance after disasters. These types of actions and responses are more in line with the concept of governability that has gained interest over the last several years, particularly in Latin American countries. Governability refers to a state of dynamic equilibrium with a multidimensional character and emphasis on the construction of a legitimate and effective response between the market, state/government, and the demands of the civil society [17].

### Disaster Governance in Urban Environments

Although Disaster Governance includes a broad spectrum of variables in its definition, the focus of this article is the study Disaster Governance through the spatial analysis of different human settlements structures in the event of a tsunami, to further our understanding of Community Resilience. In this respect, specific studies suggest that Disaster Governance in human environments can be characterized by three attributes: redundancy, overlap, and diversity [2,3,4,5,6,7,8,9,10,11,12,13,14,15,16,17,18,19,20,21]. Firstly, the redundancy of the governance structure [2] indicates that if one organization fails to provide emergency support to a struggling community, another organization similar in nature may take its role. For instance, if hospitals are damaged after a disaster, institutions such as the Red Cross and local clinics can continue providing health services to the community. Secondly, overlap in governance refers to the set of linked capacities that allow institutions to be efficient and flexible [18]. Overlap in governance develops when different types of institutions (e.g., police, firefighters, and health services among others) have the capacity to perform the same function at different scales when disaster arises [19,20]. For example, if hospitals are damaged, other institutions, such as firefighters, should be prepared to provide health assistance until the primary health support has recovered. Thirdly, diversity in governance refers to the diversity of institutions prepared to perform in the same areas, regardless of their role. For instance, a community with overlap and redundancy in governance may have a more diverse response to disaster [21]; thus, disadvantaged and threatened societies can have improved Community Resilience.

Various studies support the idea that specific aspects of urban structure and space affect the attributes of Disaster Governance. Communities with polycentric urban structures show more overlap and redundancy in governance [19]. Specifically, urban areas with various central areas often have similar neighborhoods, mixed-use areas, and modular systems [19]. For instance, in polycentric urban areas, several fire departments are distributed within different neighborhoods; therefore, if one fails, another can fill its role. Additionally, more intense overlap has been found in the central area of cities, and this overlap diminishes toward the periphery and rural areas [22]. As a result of being centrally located or due to infrastructure that facilitates mobility (e.g., properly marked paved streets), central urban areas are usually better equipped than rural and peripheral areas thus facilitating rapid and effective emergency responses. In contrast, peripheral and rural areas, which are sometimes harder to reach, often receive less assistance during an emergency. Furthermore, diversity of governance can be improved by attributes of the urban space. A diverse set of green areas with different sizes and spatial arrangements provides more opportunities for different emergency institutions to fulfill their roles after a disaster (e.g., for allocating temporary hospitals, refuges, etc.) [23]. The multifunctional use that a diversity of green areas provides contributes to the diversity of governance by providing flexibility in the urban space [24]. Diversity has also been evaluated by measuring urban density, a variable that at the same time influences the abundance/scarcity of useful open space [25]. In this case, increased urban density is associated with less diversity because there is less open space available for emergency purposes.

This study is aimed at furthering our understanding of Community Resilience through the lens of Disaster Governance in coastal environments. To do so, we have explored how different human settlement structures influence the performance of emergency institutions after disaster. This study was undertaken in four coastal towns in southern Chile that are threatened by tsunamis. The four towns selected have different spatial and planning arrangements. The objective was to evaluate, by means of different spatial indexes, the extent that spatial aspects of towns impact the attributes of Disaster Governance, namely redundancy, overlap, and diversity. The outcomes of this study can provide guidelines for improving Community Resilience through planning.

## 2. Materials and Methods

This study employed a comparative approach including three steps. First, it was necessary to identify where emergency institutions go and how they use urban spaces in the case of a tsunami emergency. For this purpose, experts in emergency procedures from different institutions were interviewed using the Projective Mapping Technique. From interviews, we identified sites used for emergency purposes. Secondly, the data were subjected to content and GIS analysis to explore how institutions are distributed and concentrated. From this, we identified the redundancy, overlap, and diversity of the institutions. Finally, the results were projected on maps and were contrasted with aspects of the spatial and planning structure of the towns, i.e., density, sector types (urban, rural or indigenous), level of government within the commune, and emergency plans, to explore their influence on disaster governance.

### 2.1. Towns of Study

**Figure 1:**
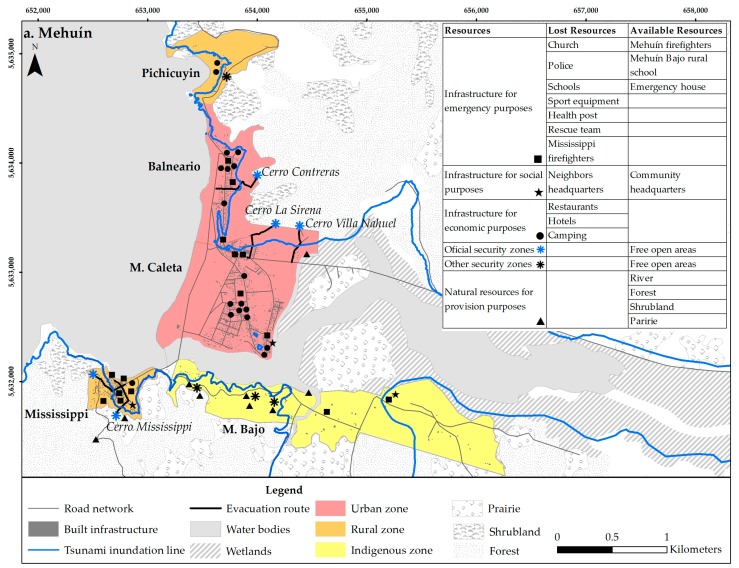

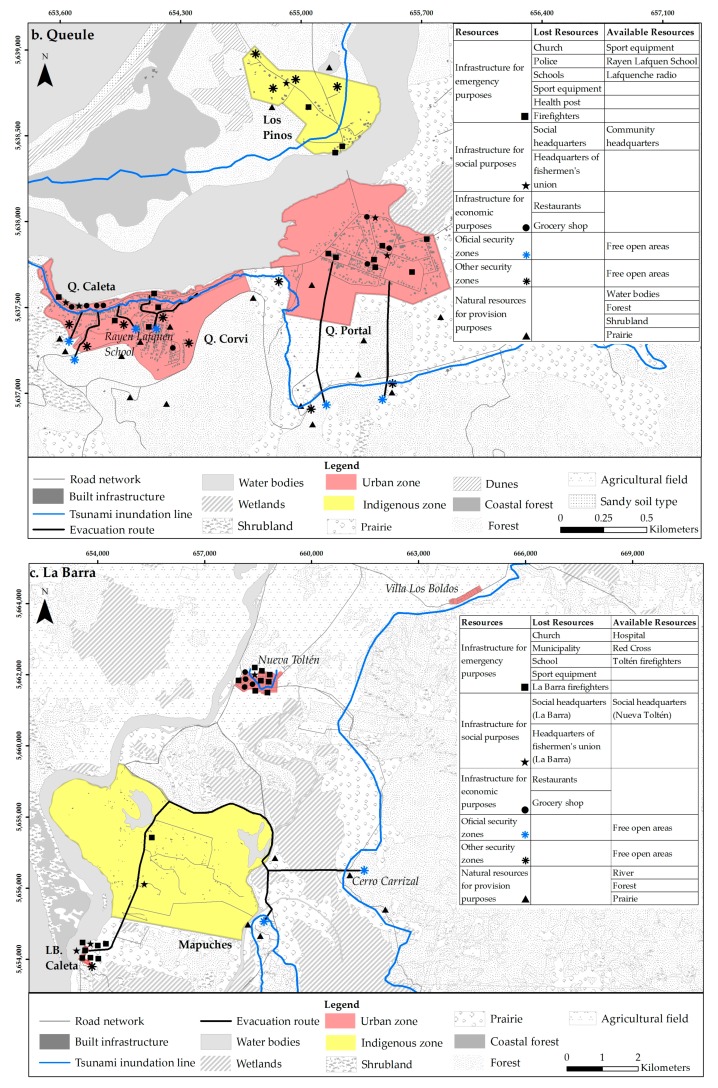

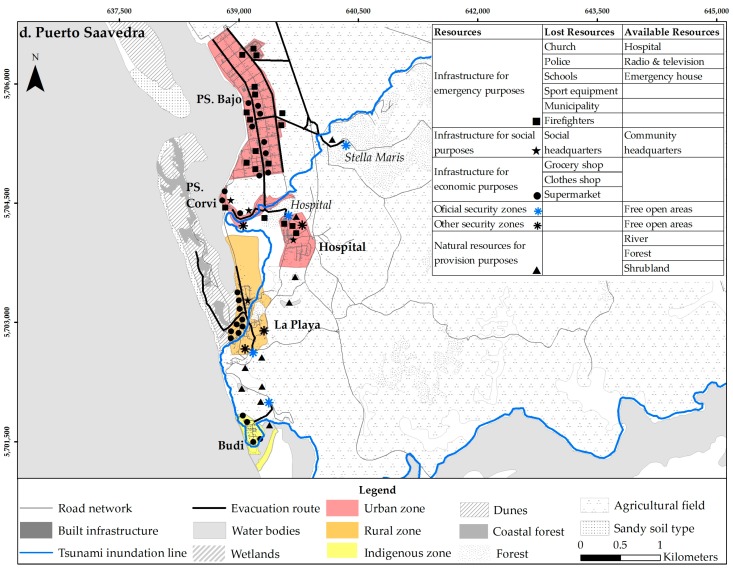
(**a**) Study town of Mehuín; (**b**) Study town of Queule; (**c**) Study town of La Barra; (**d**) Study town of Puerto Saavedra. Note: These maps show the urban, rural and indigenous areas of each study town, and indicate official security zones and evacuation routes. In addition, the available and lost resources after tsunami are mapped considering their location below and above the tsunami inundation line [26]. Sources: Civil Protection Plan in case of Tsunami 2013, Land Use Plans of Saavedra 2013 and Toltén 2012, and fieldwork on site.

The study areas included four towns distributed within three communes and two regions in southern Chile (Figure 1). While Mehuín, Queule and La Barra represent the lowest units of government, Puerto Saavedra represents a higher unit, as it is the capital of the Saavedra Commune. All towns have a monocentric structure with urban, rural, and indigenous sectors. Additionally, all towns have tsunami emergency plans. Mehuín is a small coastal town with a population of 1504 inhabitants, and it is located in the Mariquina Commune in the Los Ríos Region. Mehuín faces the sea, lies at the mouth of the Lingue River, and includes the urban sectors of M. Caleta and Balneario, the rural sectors of Pichicuyin and Mississippi, and the indigenous sector of M. Bajo. Queule is also a small coastal town with a population of 1541 inhabitants located in the Toltén Commune in the La Araucanía Region. It is located along both sides and at the mouth of the Boldo River and includes the urban sectors of Q. Corvi, Q. Caleta, and Q. Portal and the indigenous sector of Los Pinos. The town of La Barra is a small fishing town of 165 inhabitants located at the mouth of the Toltén River in the Toltén Commune in the La Araucanía Region. La Barra includes the urban sector of LB. Caleta by the coast and the indigenous sector of Mapuches located inland. Finally, Puerto Saavedra is a large coastal town of 5011 inhabitants located in the Saavedra Commune. It is the capital city of the commune and is separated from the sea by the Imperial lagoon. Puerto Saavedra includes the urban sectors of PS. Bajo and PS. Corvi, the rural sector of La Playa, and the indigenous sector of Budi.

### 2.2. Data Collection and Participants

**Figure 2:**
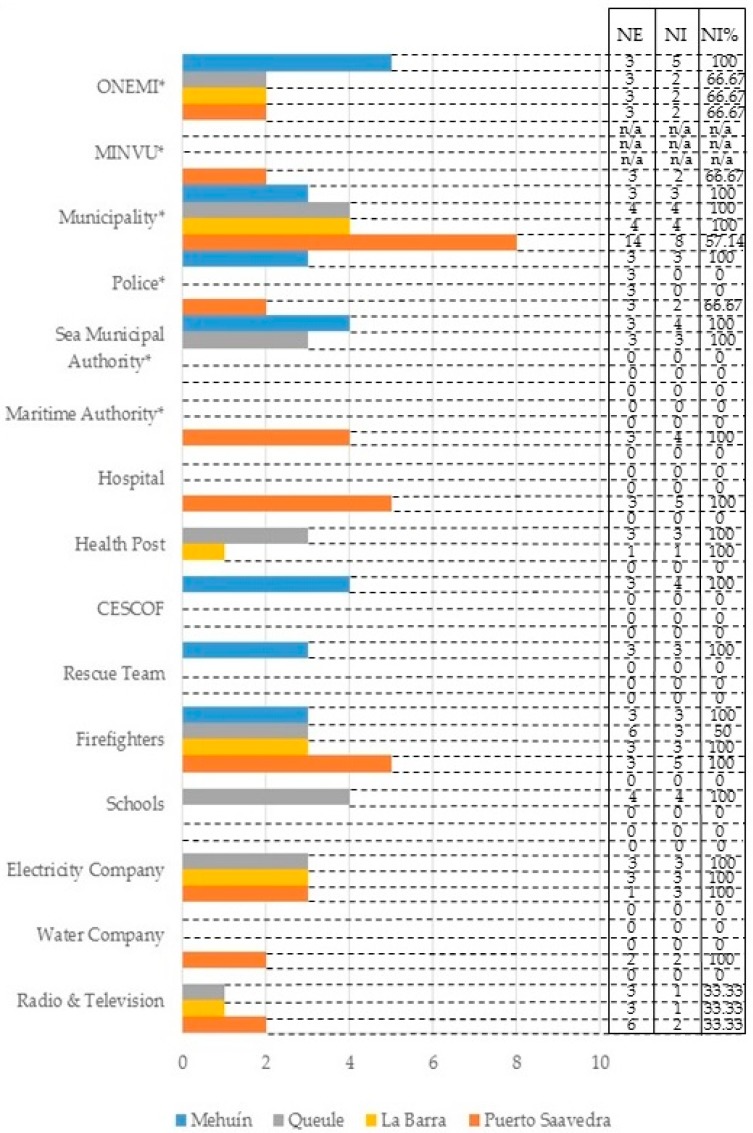
Percentage of people interviewed from each emergency institution. Note: ONEMI stands for the National Emergency Office. MINVU stands for the Ministry of Housing and Urbanism. CESCOF stands for Family Health Center. NE stands for Expected number of interviews. NI stands for number of interviews undertaken. NI% stands for the percentage of number of interviews undertaken. The * indicates governmental institutions.

Participants from government and non-governmental institutions who are involved in tsunami emergency procedures were invited to participate in an interview. The participants contacted were identified based on the information provided by the local Emergency Operation Committees (COE) of each commune; the COE is in charge of the management of disaster situations. Institutions are distributed in each town as can be observed in Figure 2. In Mehuín, a total of 25 professionals from four government institutions and three non-government institutions were interviewed. In Queule, a total of 23 participants from four government institutions and five non-governmental institutions were interviewed. In La Barra, a total of 15 participants from two government institutions and four non-government institutions were interviewed. Lastly, in Puerto Saavedra, a total of 35 participants from five government institutions and five non-governmental institutions were interviewed. Overall, a total of 32 institutions and 98 emergency professionals (75.96% of the population) were interviewed. The role of each participant during an emergency is described in Table 1, including the coordination of the emergency response, evaluation of damages, evacuation, medical care, and the distribution of supplies, among others. Using the Projective Mapping Technique, participants were interviewed in their own workspaces. The interviews consisted of showing participants a map of the town under study and asking them to identify the places they would use during the emergency stage of a tsunami to provide professional help to the community. Participants were also asked to mention the utility of the places (e.g., for refuge, for water collection, for temporary health service). With this information it was possible to identify the frequency of mention of sites and the site uses for emergency purposes in the event of a tsunami.

### 2.3. Content Analysis

Content analysis [27] was used to reduce the number of uses assigned by participants to the places used for emergency purposes to a manageable number. The analysis involved the following steps to assure validity. First, uses with the same meaning were grouped together (e.g., the terms “for water collection” and “for water extraction” were grouped under “water supplies”). Second, the outcome of the first step was group together by theme (e.g., “water supplies” and “food supplies” were grouped under “supplies”). Accordingly, the themes represent the final number of uses assigned by participants to the sites used for emergency purposes. This procedure was performed twice and by two researchers separately to assure that the analysis was reliable and trustworthy [28].

### 2.4. GIS Analysis and Spatial Index

Frequency data were organized in different matrices (m × n) prepared to code the mention of sites (ID × n) and frequency of mention of uses (ID × Use). In the first case, the number 1 was used when a site was mentioned by each organization; otherwise, the number 0 was assigned. In this manner, four binary matrices were created, one for each town, and were subjected to the Directional Distribution Index. The Directional Distribution Index was used to evaluate the territory that is under the jurisdiction of each institution; this index was used to determine the redundancy and overlap of each institution in the event of a tsunami emergency. This technique was originally developed for the ecological study of animal habitats and consists of obtaining a bivariate confidence interval for X and Y coordinates. Once the confidence intervals were determined, the standard distances (Euclidian) between the X and Y locations of the sites were calculated. These distances were then mapped to show the ellipsoid area of the sites mentioned by each emergency institution. The X and Y coordinates define the major and minor axes of the ellipse with the smallest possible area. Each standard deviational ellipse is a summary of the data dispersion of a point structure. The size of the ellipses depends on the standard deviation. In this case, a standard deviation of 1 covered 68% of the places mentioned. Results can be interpreted as follows: increased redundancy is shown by more ellipses representing institutions with similar roles and crossing in the same sector. Likewise, there is more overlap in governance if there are more ellipses representing different institutions prepared to assume similar roles in the same sectors.

The frequency of mention of sites was recorded for each use associated to each place mentioned by the participants. Accordingly, four frequency matrices were obtained for each town. These were then subjected to the Kernel Density Index. The Kernel Density Index was used to identify the concentration of site uses and the most used sites in the towns. This was used, hence, to shed light on the multifunctionality, or diversity, of the sites. Kernel Density refers to how a phenomenon is spread across a landscape based on the quantity of a variable, for example evacuation, which is measured at each point. This analysis is based on the quadratic kernel function and can be used to explore spatial variation in event intensity [29,30]. Specifically, this index was used to calculate the uses associated with sites and their surrounding area. Thus, the analysis showed the density of 1 km^2^ sites mentioned by institutions; these were represented by five ranges for the four towns. The pixel size used was 25 × 25 m, including a spherical surface and a radius of 200 to 500 m. The results are illustrated in maps, which indicate the urban nodes with more intensity of use. The Kernel Density values are differentiated according to the shade and intensity of a particular color. Accordingly, the resulting kernel raster values are greater when the color is more intense, indicating that more points are located in the area. Overall, higher raster values reflect more diversity in governance, suggesting that more institutions are using the space for emergency purposes.

## 3. Results

### 3.1. Site Types and Uses

**Figure 3:**
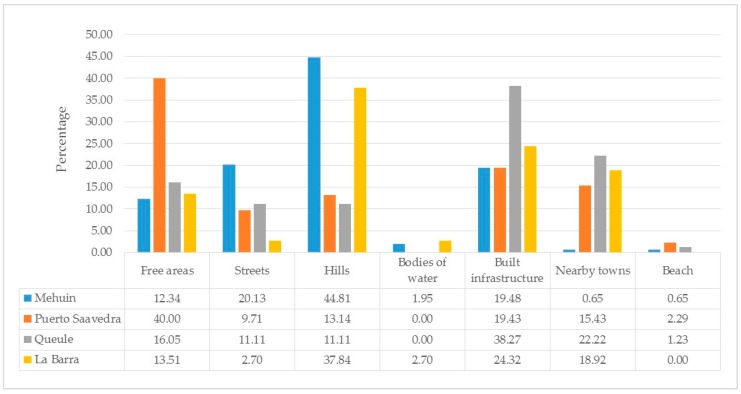
Frequency of mention of site typologies for each town.

After the application of the Projective Mapping Technique, 29 sites were identified for Mehuín, 30 for Puerto Saavedra, 34 for Queule, and 16 for La Barra. These were classified into the following seven typologies using the content analysis approach: (1) “Free areas”, such as area for sports, open spaces, stadiums, coves, viewpoints, and safe areas; (2) “streets”; (3) “hills”; (4) “bodies of water” including rivers, estuaries, water springs, wetlands, and ravines; (5) “built infrastructure”, such as schools, hospitals, fire stations, gymnasiums, churches, radios, faucets, rafts and community venues; (6) “nearby towns”; (7) and “beach” (Figure 3). The most frequently mentioned site typologies were built types. These included “built infrastructure” in all cases (Mh = 19.48%, PS = 19.43%, Ql = 38.27%, LB = 24.32%) and “nearby towns” in most cases (PS = 15.43%, QL = 22.22%, LB = 18.92%). Also, “hills” were often frequently mentioned in Mehuín (44.81%) and La Barra (37.84%), and “free areas” were commonly mentioned in Puerto Saavedra (40%). On the contrary, the typologies less frequently mentioned were those belonging to natural environments. These included “bodies of water” (Mh = 1.95%, PS = 0%, Ql = 0%, LB = 2.7%) and the “beach” (Mh = 0.65%, PS = 2.29%, Ql = 1.23%, LB = 0%).

**Figure 4:**
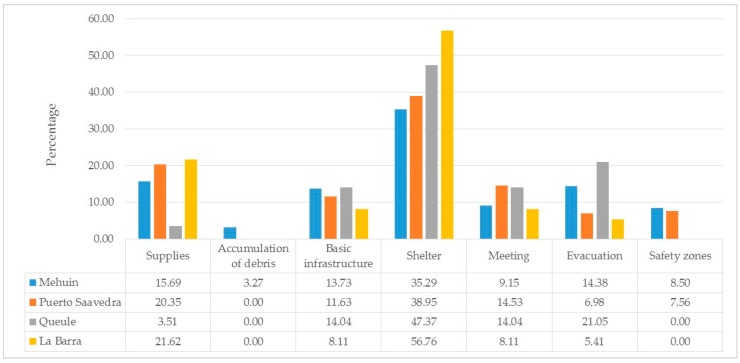
Frequency of mention of uses for each town.

Emergency professionals assigned 20 different uses to these sites, and with the application of content analysis explained in Section 2.3, these were reduced to seven. Figure 4 shows the outcomes of the content analysis indicating that emergency sites can be used for: (1) The distribution of “supplies”, including water, food, and firewood; (2) the “accumulation of debris” such as that coming from destroyed infrastructure; (3) the implementation of “basic infrastructure” such as for cooking and health care; (4) the implementation of “shelters” including temporary and permanent closed areas where people can take refuge until their houses are repaired; (5) the holding of “meetings” that involve the dissemination of information about the extent of the disaster, the actualization of emergency procedures, and activities for community recreation; (6) the process of “evacuation” including the network of streets that provide access to safety zones; (7) and the allocation of “safety zones” that keep the community safe in the event of a tsunami. The use of sites for “shelter” was the most frequently mentioned in all towns (Mh = 35.29%, PS = 38.95%, Ql = 47.37%, LB = 56. 76%), while the “accumulation of debris” (Mh = 3.27%, PS = 0%, Ql = 0%, LB = 0%) and “safety areas” (Mh = 8.5%, PS = 7.56% , QL = 0%, LB = 0%) were mentioned the least and were not declared at all in some cases. This does not mean that one is more important than the other. The results suggest that there are certain uses that are more likely to be implemented in more sites than others, yet all uses are relevant to the community.

### 3.2. Directional Distribution Index

**Figure 5:**
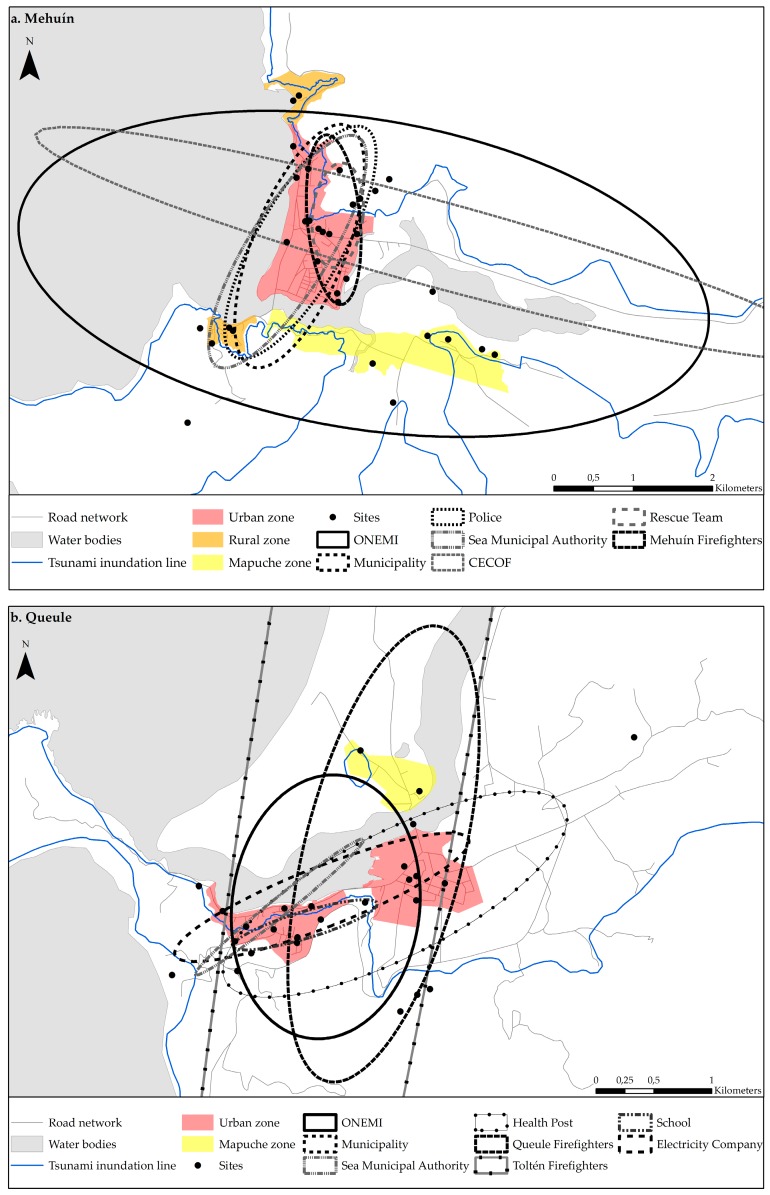

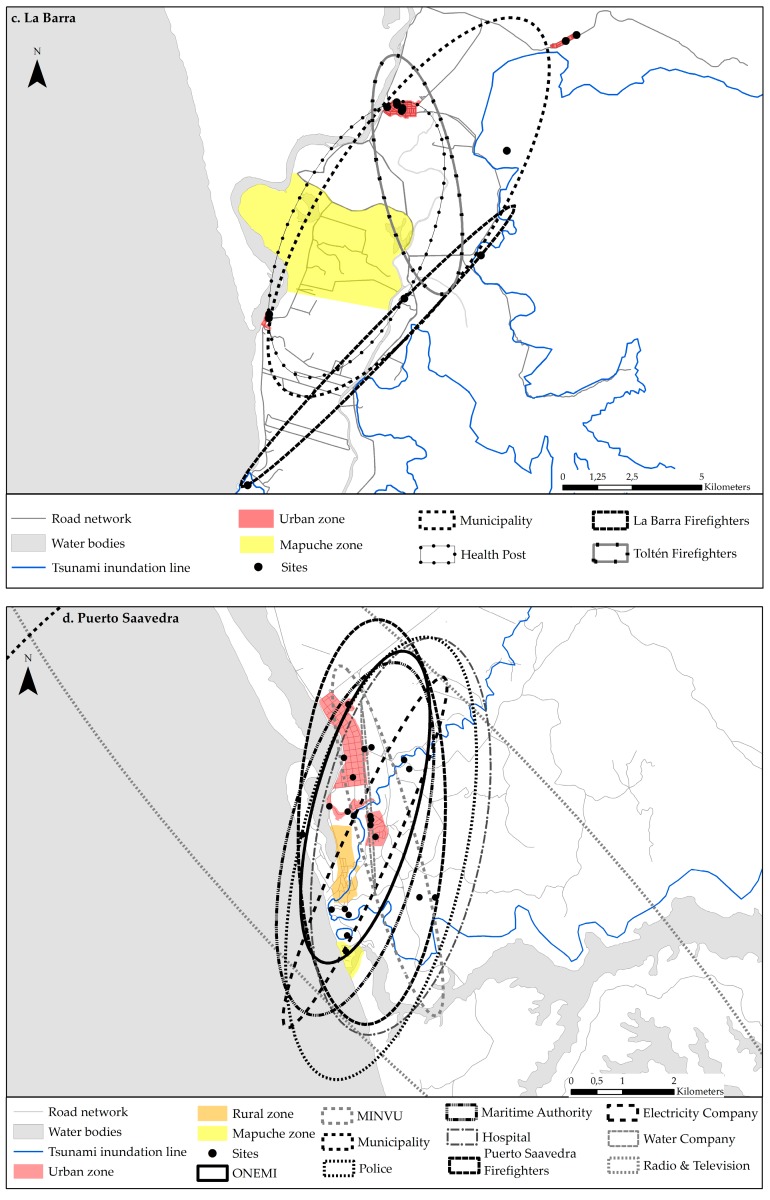
(**a**) Directional Distribution Map: Mehuín; (**b**) Directional Distribution Map: Queule; (**c**) Directional Distribution Map: La Barra; (**d**) Directional Distribution Map: Puerto Saavedra.

The results indicated that in the case of Mehuín, the ellipses have a predominant north-south direction and mostly cover urban sectors (Figure 5a, Table 2). The crossing of all of the ellipses occurred in the urban sector of M. Caleta, at the security area located 30 m.a.s.l. near the fire department, and at a school used as shelter. The ONEMI ellipse was the largest. Surpassing the town limits, this ellipse had an area of 26.98 km^2^ in an east-west direction and with an angle of rotation of 99.47. In contrast, the Rescue Team ellipse was the smallest and had an area of 0.66 km^2^. The Rescue Team ellipse lied in a north-south direction and had an angle of rotation of 5.79. The sectors where there was the least amount of ellipse crossings were mainly areas outside the urban boundary and/or without emergency infrastructure. The rural sector of Mississippi was only covered by the ONEMI, Municipality, Police, and the Sea Municipal Authority ellipses (four out of seven). Additionally, the indigenous sector of M. Bajo was only covered by the ONEMI ellipse. The rural sector of Pichicuyin was not found to be covered by any emergency organization.

The results for Queule were similar to those found for Mehuín. The ellipses mainly cover the urban center and to a lesser degree covered the rural and indigenous sectors (Figure 5b, Table 2). The Municipality ellipse was the largest with an area of 217.28 km^2^. This ellipse lied in a north-south direction and had a rotation angle of 9.79. In contrast, the Sea Municipal Authority ellipse was the smallest. It had an area of 0.23 km^2^ and lied in east-west direction with an angle of 51.12. The sector that was covered by the largest number of ellipses (seven out of eight) was Q. Corvi, a touristic center located 30 m.a.s.l. with housing and educational infrastructure. In Q. Corvi, the ONEMI, School, Queule Firefighters, Toltén Firefighters, Electricity Company (SAESA), Health, and Municipality institutions interact (seven out of eight). Furthermore, the Rayen Lafquen School is located in this sector and serves as the official emergency shelter. Contrary to that found in Q. Corvi, the indigenous Los Pinos sector was only covered by the Toltén Firefighters, Queule Firefighters, and the Municipality ellipses (three out of eight).

In the case of Puerto Saavedra, the direction and size of the ellipses were similar and mainly crossed over the urban sectors (Figure 5d, Table 2). The predominant direction of the ellipses was from north to south. The Municipality ellipse was the one of the largest with a surface area of 294.81 km^2^ and with a west direction and an angle of rotation of 68.71. Contrarily, the Aguas Araucanía ellipse was the smallest with 0.23 km^2^. This small ellipse lied in a north-south direction and had an angle of 175.91. Within the urban sector, the areas with the most ellipse crossings were located 30 m.a.s.l. and corresponded to the hospital sector and the Stella Maris evacuation site. The hospital sector, including housing and health infrastructure, was covered by nine out of ten ellipses, excluding the Water Company ellipse. The Stella Maris site is the only well-equipped evacuation site in Chile. Eight out of ten ellipses crossed at the Stella Maris site; these excluded the Water Company and MINVU ellipses. In the rural La Playa sector, eight out of ten ellipses interacted; these excluded the Water Company and the MINVU ellipses. The sector with the least ellipse crossings (six out of ten) was Budi, which is rural. The ellipses crossing in Budi excluded the Water Company, Electricity, ONEMI and MINVU ellipses.

The results for La Barra were radically different from those of the other towns. The predominant direction of the ellipses was from north to south (Figure 5c, Table 2). The Municipality ellipse was largest, with a surface area of 74.03 km^2^; this ellipse lied in an east-west direction and had a rotation angle of 33.72. The smallest ellipse was the La Barra Firefighters ellipse with a surface of 11.72 km^2^ and lying in an east-west direction with a rotation angle of 44.21. The most ellipse crossings occurred in the town of Nueva Toltén. This town is 11 km from La Barra and is the capital of the commune. Unlike La Barra, Nueva Toltén is equipped with emergency infrastructure. In Nueva Toltén, the Municipality, Toltén Firefighters, and Health Post ellipses interacted (three out of four). The same three ellipses also intersected at Cerro La Piedra, a hill that is within a Mapuche indigenous sector, five kilometers from LB. Caleta and 30 m.a.s.l. This hill has been historically known as an evacuation point during tsunami events. The smallest number of ellipse crossings occurred at LB. Caleta, which is located in a tsunami inundation zone. In LB. Caleta, only the Municipality and the Health Post ellipses intersected (two out of four).

### 3.3. Kernel Density Index

The results of the Kernel Density analysis indicated the sites with the greatest intensity of use. Thus, higher values suggest sites in which a greater diversity of institutions interact. In the towns studied, sites included “hills”, “built infrastructure”, “free areas” and “nearby towns”. In all locations it was found that the sites with less diversity of use were natural spaces such as “bodies of water” and “beaches”. These natural sites were often located in rural and indigenous sectors and lacked emergency infrastructure.

**Figure 6:**
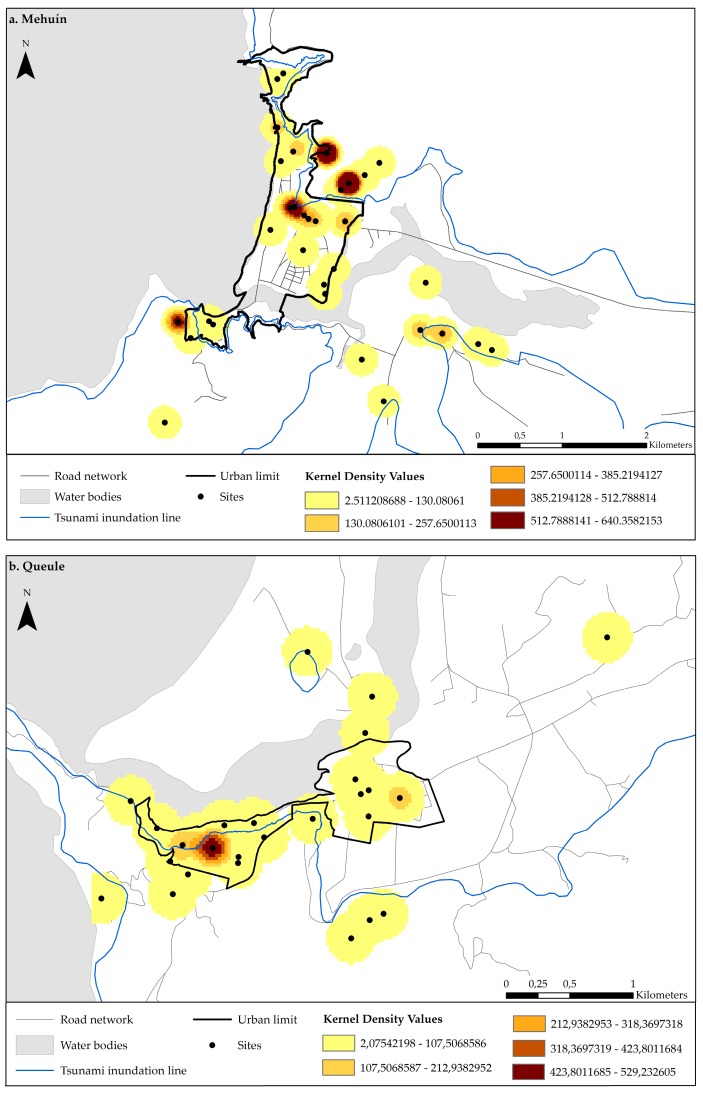

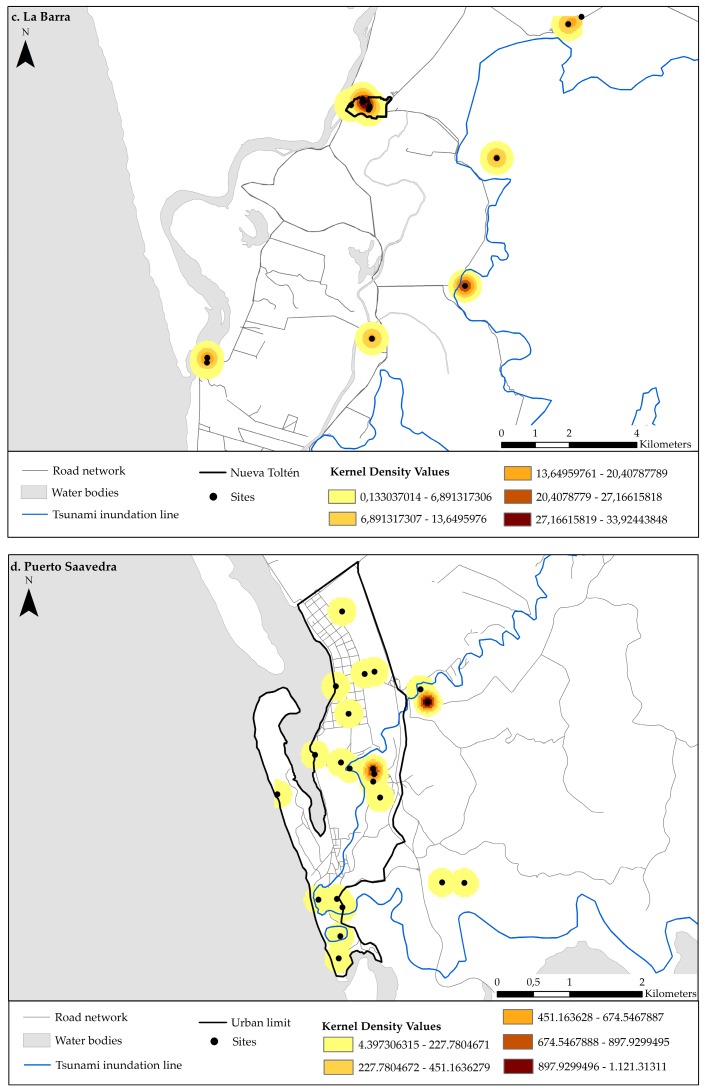
(**a**) Kernel Density Map: Mehuín; (**b**) Kernel Density Map: Queule; (**c**) Kernel Density Map: La Barra; (**d**) Kernel Density Map: Puerto Saavedra.

For Mehuín, the densities varied between a minimum of 2.51 and a maximum of 640.35 (Figure 6a). Four sites, typologically classified as “hills”, were found to be used most intensely (Cerro La Sirena, Cerro Contreras, Cerro Villa Nahuel, and Cerro Mississippi) (Figure 1). These “hill” sites are characterized by being 30 m.a.s.l. and without infrastructure. According to the results, emergency institutions would use these hills for “shelter”, (Cerro La Sirena = 37.5%, Cerro Contreras = 44.44%, Cerro Villa Nahuel = 33.33%, Cerro Mississippi = 35.71%), “basic infrastructure” (Cerro La Sirena = 25%, Cerro Contreras = 14.81%, Cerro Villa Nahuel = 25%, Cerro Mississippi = 7.14%), “supplies”, (Cerro La Sirena = 12.5%; Cerro Contreras = 18.52%; Cerro Villa Nahuel = 16.67%; Cerro Mississippi = 28.57%), “accumulation of debris” (Cerro La Sirena = 12.5%, Cerro Contreras = 3.7%, Cerro Mississippi = 14.29%), “evacuation” (Cerro Contreras = 3.7%, Cerro Villa Nahuel = 4.17%, Cerro Mississippi = 7.14%), “meeting” (Cerro Contreras = 7.41%, Cerro Villa Nahuel = 12.5%, Cerro Mississippi = 7.14%) and as a “safety zone” (Cerro La Sirena = 12.5%, Cerro Contreras = 7.41%, Cerro Villa Nahuel = 8.33%).

The other towns had fewer sites with high intensity of use. In Queule, site index values ranged from 2.07 to a maximum of 529.23 (Figure 6b). The results indicated that there was only one site, the Rayen Lafquen School, with a high intensity of use. This site is classified as “built infrastructure”, and the site is predominantly used as “shelter” (63.64%), “basic infrastructure” (22.73%) and for “meetings” (13.64%). For La Barra the density values ranged from a minimum of 0.13 to a maximum of 33.92 (Figure 6c). Three sites had high intensities of use. These sites were classified as having “hill” (Cerro Carrizal) and “nearby towns” (Nueva Toltén and Villa Los Boldos) typologies. The uses assigned to these sites included “supplies” (Cerro Carrizal = 28.57%, Nueva Toltén = 66.67% and Villa Los Boldos = 100%) and “shelter” (Cerro Carrizal = 71.43%, Nueva Toltén = 33.33%). Finally, two sites, Stella Maris (“built infrastructure”) and the Hospital (“free area”) in Puerto Saavedra were found to have high usage intensities (Figure 6d). These sites are 30 m.a.s.l and both have indoor and outdoor space useful for emergency procedures. The uses assigned to these sites were “shelter” (Stella Maris = 42.55%, Hospital = 25%), “supplies” (Stella Maris = 34.04%, Hospital = 14.29%), “meeting place” (Stella Maris = 10.64%, Hospital = 21.43%), “basic infrastructure” (Stella Maris = 6.38%, Hospital = 32.14%) and “safety zone” (Stella Maris = 6.38%; Hospital = 7.14%). The density values for Puerto Saavedra ranged from 4.39 to a maximum of 1121.31.

## 4. Discussion

### 4.1. Overlap in Governance, Town Structure, and Planning Instruments

**Figure 7:**
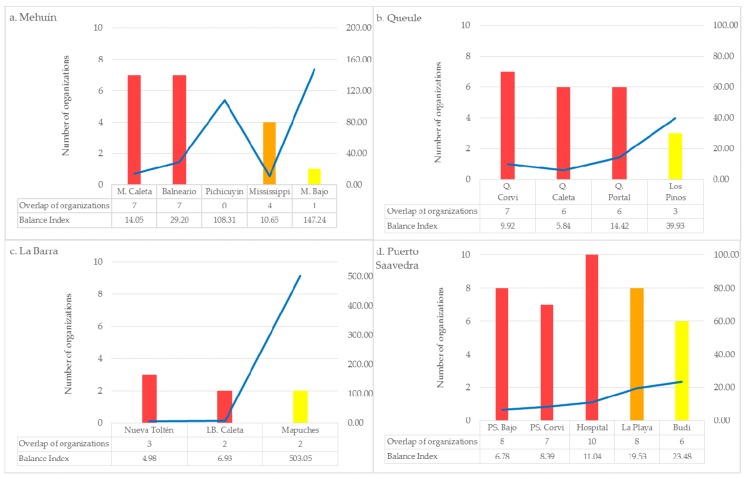
Overlap in governance and balance index. Note: Red, orange, and yellow colors are used to indicate urban, rural, and indigenous sectors respectively.

The results of the directional distribution indicator analysis were interesting because the ellipses served as proxies of the area covered by the emergency institutions. First, the area where the ellipses crossed indicated the spatial coverage of the institutions in the event of an emergency. The interpretation of results shown in Figure 7 illustrates that most of the ellipses overlap in the central sectors of the towns. Conversely, there was less ellipse overlap in the rural and indigenous sectors located near the periphery of the towns. The comparison of graphs in Figure 7 indicates that the same situation is observed in all of the towns.

In order to explain this finding, the Balance Index [31] was used to compare densities between centric and peripheral areas. This index is a measure of the balance between built and unbuilt areas; the lower the index, the more built areas. Built infrastructure includes not only emergency buildings but also public buildings, services, and housing. We have found considerably higher values of this index in the rural and indigenous sectors located in peripheral areas where unoccupied space is abundant (Figure 7). Furthermore, urban density is higher in the central parts of the towns due to more built space. Mississippi, located in Mehuín, is an exception to this finding because it is currently undergoing urbanization in order to become urban in the near future. Previous studies have found less overlap in governance in denser areas, and this has been explained by the lack of open space that can be used for the application of emergency procedures [22]. In this study, the variation in overlap in governance is also explained by the agglomeration of infrastructure (emergency infrastructure in particular) in centric and urban sectors.

Villagra and colleagues [22] observed that the center and the periphery of towns strongly differ in terms of their ability to adapt after a disaster. This seems to also hold true for the study herein presented. In the town centers there was increased overlap of institutions compared to that found towards the periphery of the towns. This indicates that the town centers have high resilience as suggested in previous studies [14,18]. The lack of disaster relief institutions in rural and indigenous areas is worsened due to the monocentric condition of the towns under study. Other studies have found that monocentric urban areas are less adaptive after disasters compared to polycentric cities [19]. Furthermore, the categorization of the territory as urban, rural, or indigenous hinders the possibility to increase institutional overlap in rural and indigenous areas. For instance, land use plans are not available for rural and indigenous sectors. As such, it would be difficult to improve the allocation of built infrastructure and land use that would be useful in an emergency and that could easily reach these more peripheral areas. Transitions zones between all areas—which are currently nonexistent—could also be helpful if they are sufficiently equipped with emergency facilities that would provide emergency services to all areas.

Nonetheless, the crossover of ellipses representing emergency response institutions can be interpreted as overlap in governance. Accordingly, Figure 7 provides evidence of overlap in governance because at least two institutions cover most sectors (with the exception of Pichicuyin and M. Bajo, which are facilitated by only one institution). Despite this, not all institutions are prepared to provide all emergency services (refer to Table 1). For example, the planning and coordination of activities during an emergency are only allocated to one institution, ONEMI, hindering overlap in governance. On the other hand, various institutions share similar roles (e.g., evacuation), which can facilitate overlap in governance; however, their performance should be carefully coordinated so that a response does not turn into chaos. For instance, different institutions might assist and provide resources in the same area at the same time, and subsequently help in other areas of the city will be delayed.

Nowadays, hazards are complex events which do not respect borders and that require extensive cross-national and cross-institutional collaboration [6]. This puts coordination at the core of the challenge of Disaster Governance because it involves actions carried out by a variety of local, national, and international entities including emergency institutions, civil institutions, and NGOs. An alternative approach called Adaptive Governance [15] has been proposed in order to govern such complexity. Adaptive Governance consists of creating a Multi-stakeholder platform (MSP), which includes a multitude of organizations at different scales of governance. The MSP then provides intellectual and financial aid and works to improve the coordination to a response. The MSP empowers stakeholders to solve a similar problem. The implementation of a MSP is supported by the concept of Governability [17] introduced in the first section, which encourages the participation of all stakeholders, particularly in developing countries. In the study herein, the COE (Emergency Operation Committee) in charge of emergencies mostly involves emergency institutions and its actions and members are restricted to one level of government (i.e., communal, regional, or national) instead of involving multiple levels of government and stakeholders, as suggested by the MSP. Furthermore, in order to insure that there is a coordinated overlap in governance in all areas of towns, planning and emergency instruments should be flexible to allow various institutions to take on alternative roles in the event that the primary organization fails. Indeed, Gall [7] states that flexibility is the one attribute that contributes to disaster governance when the adjustment of policies and regulations is necessary in an emergency situation. Flexibility requires preparation and the allocation of resources. This is particularly relevant for the towns studied here.

### 4.2. Redundancy in Governance and Regional Structure

**Figure 8:**
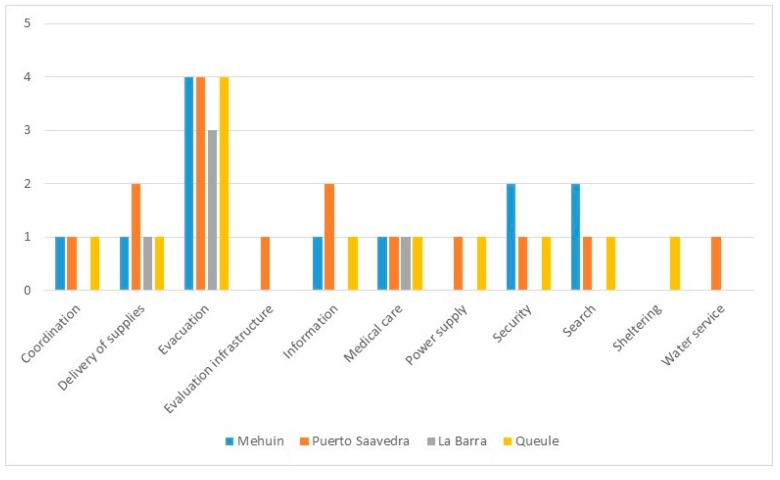
Distribution of the number of institutions with similar roles in each town.

Results of the Directional Distribution analysis also provide information regarding redundancy in governance. In this analysis, redundancy in governance can be interpreted as the crossover of ellipses representing institutions of similar nature. Accordingly, redundancy in governance was found in all of the towns studied; however redundancy was restricted to few emergency response roles (Figure 8).

In this study, there was a high degree of crossover of ellipses representing “evacuation” (Table 1 and Figure 8). In Mehuín, evacuation is facilitated by the Municipality, the Police, Firefighters and by the Sea Municipal Authority. In Queule, the crossover of the Municipality, Sea Municipal Authority and two different firefighter department ellipses reflects high redundancy in evacuation. In La Barra, there are also two firefighter departments involved in evacuation. In Puerto Saavedra, the Municipality, the Police, Firefighters and the Maritime Authority provide “evacuation” support in the Hospital sector. Maritime Authority institutions, only found in Puerto Saavedra, are usually located in towns of higher communal hierarchies, such as Puerto Saavedra, which is the capital of the commune. The Maritime Authority has several other roles during a tsunami emergency such as that of reporting information, searching for missing persons, registering damage, and distributing supplies. These needs can also be provided by the Sea Municipal Authority, Municipality, Rescue Team, Health post, Firefighters, MINVU and Radio and Television services (Table 1). Thus, having the Sea Municipal Authority in town increments redundancy of governance, which, in turn, adds to the adaptive capacity of the town.

It is interesting to observe from these results that redundancy in governance depends not only on the type of institutions involved in emergency responses but also on the level of government of the towns within the communes and regions. In the case of Queule and La Barra, which represent the smallest units of government according to the governmental arrangements, redundancy is, in part, due to the fact that both, La Barra and Queule have their own fire departments apart from the communal fire department. In the case of Puerto Saavedra, which is of a higher level of government within the commune, the presence of the Maritime Authority enhances redundancy. Apart from the support in evacuation, the Maritime Authority together with the Municipality can help deliver supplies and provide information in collaboration with the TV station, which is also located in Puerto Saavedra.

These findings reflect those found by Gall [7] regarding the importance of cooperation and collaboration at a variety of scales for effective disaster governance. Accordingly, responses to an emergency can be distributed among similar institutions within and also nearby the disaster area. In this way, cross-regional and cross-organizational collaborations can be facilitated [6]. Such cooperation is particularly important in developing countries, where there are great disparities in income, well being, and access to services among human settlements [14]. In our study, an example of this is the town of La Barra, which includes only a fire department, community house, and a health post. All other regional institutions involved in emergency procedures such as the police department, schools, and water and electricity companies are located in Nueva Toltén. This explains why Nueva Toltén, located 11 km from La Barra, is where most of the emergency procedures occur. Nonetheless, La Barra has redundancy of evacuation due to the flexible role of the local and communal firefighter department. Additionally, this town and has the potential to have more redundancy in governance. For instance, if the jurisdiction of the Hospital of Nueva Toltén is flexible, this hospital could perform health assistance on site in La Barra in the event that the Health Post of La Barra fails after a disaster.

### 4.3. Diversity in Governance and Emergency Plans

**Figure 9:**
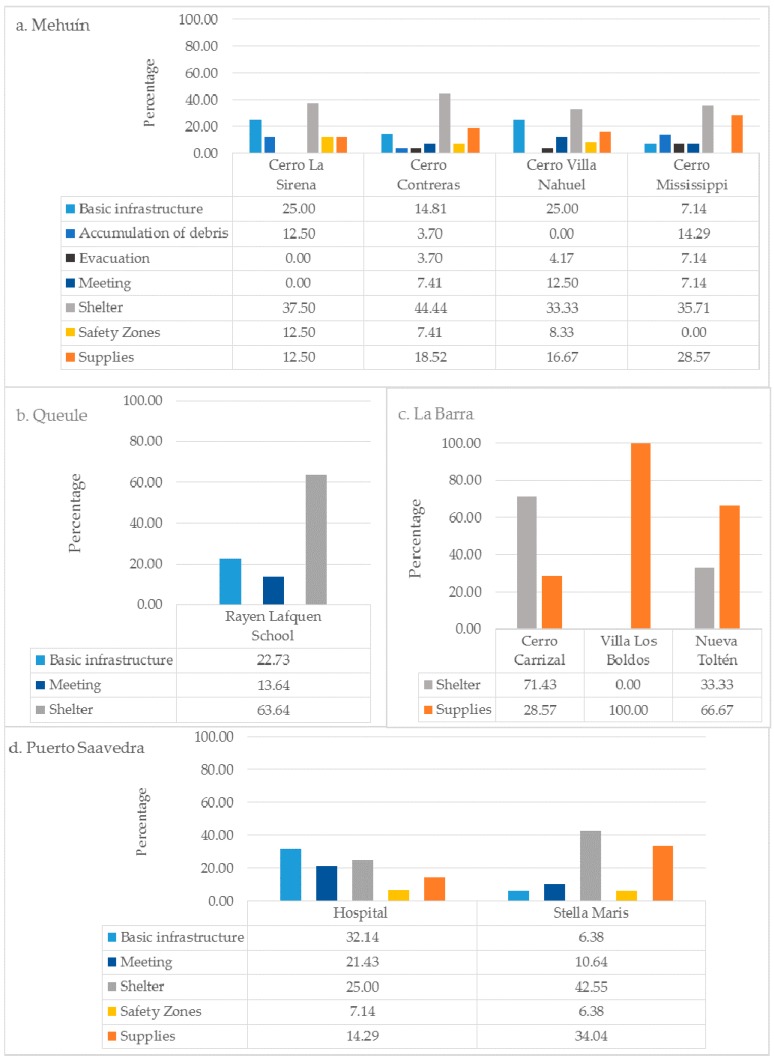
Sites with high diversity in governance.

Diversity in governance is an aspect of resilience that influences the adaptive capacity of cities to respond to disasters [25,32]. Here, the application of the Kernel Density index indicates that sites that are prepared to host a diversity of uses during emergencies are few in each town. The results interpreted in Figure 9 indicate that in Mehuín there are four sites that are intensely used; these sites are surrounded by forest, located on hills, and without infrastructure. In Queule, there is only one site, a school, with a high intensity of use. This site is categorized as “built” and is located on a hill. In La Barra, there are three sites that are intensely used; one is located on a hill and is without infrastructure, and the other two areas are located in human settlements. Finally, in Puerto Saavedra there are two sites that are intensely used; one is categorized as “built” and is located on a hill (The Hospital), and the other site is categorized as “unbuilt” and is also located on a hill (A Lookout). Both sites have infrastructure to support an emergency response.

In addition, Figure 6 shows that sites prepared to host a diversity of uses are heterogeneously distributed within each town. The clustering of sites at higher elevations that provide refuge from rising water is expected in tsunami prone environments, as is the case here. However, other studies have reported that a more homogeneous distribution of sites facilitates resilience because it provides equal access of services to all [19,22]. Thus, this together with the results found here suggest that the communities are less resilient due to the agglomeration of intensively used sites at higher elevations.

Another common feature of the sites is that all of them are indicated in the emergency plans of each town. Indeed, they are the only sites indicated in the town plans (Figure 1). Accordingly, we can infer that diversity in governance is determined by what has been included in these plans. There may be other sites with attributes that would facilitate the interaction of emergency institutions after a disaster; however, due to the regulations established in emergency instruments, institutions mostly perform where they are instructed to provide aid. Previous studies have highly encouraged collective action to improve Disaster Governance [6]; however, in the towns studied here, this would be hindered by the limitations established in the emergency plans.

## 5. Conclusions

The study of Disaster Governance undertaken here shows that Disaster Governance is highly influenced by decisions taken during regional, urban, and emergency planning. Here it was found that towns with higher hierarchies within communal systems have better Disaster Governance. One reason for this is that most of the emergency institutions studied here are located in central and urban areas, which, in turn, have more redundancy, overlap, and diversity in governance should a tsunami occur. In contrast, emergency plans add to the limitation of governance in the towns studied. Although the emergency plans include both governmental and non-governmental institutions which altogether contribute to the diversity and redundancy of an emergency response, the roles allocated to emergency institutions are not diverse enough, neither is the distribution of safety and evacuation zones within the towns. Accordingly, and since the emergency institutions have to follow the emergency procedures established in emergency plans, flexibility-known as one of the characteristics that influences Disaster Governance [7]—cannot be achieved during an emergency.

From our findings, it is seen that Disaster Governance is improved when governmental and non-governmental institutions interact. Although the government was found to be an important institution during an emergency, it was also found that the government is insufficient by itself. Hence, governability can improve Disaster Governance by encouraging governmental and non-government institutions as well as society to interact. In support of this idea, other studies have highlighted the need for an approach that includes the interrelationships of norms, institutions, actors, and practices during an emergency (e.g., MSP approach discussed in the discussion section) [4,15,17]. In other words, governability calls for collective action not only among actors from different institutions, but also among actors and planning instruments. Further studies should include the role of the local community in responding to a disaster and explore the extent to which redundancy, overlap, and diversity in governance are improved.

Overall, the spatial relationships found between different town structures and the performance of government and non-government emergency institutions in the event of a disaster show that urban areas have better Disaster Governance than rural and indigenous areas. However, the availability of resources remaining after disturbance in the study areas, influence the spatial findings obtained in this study. Figure 1 shows that urban areas, unlike rural and indigenous areas, are better equipped with emergency facilities and infrastructure that host economic activities, yet most urban areas are located in tsunami inundation zones. This suggests that the effect of a tsunami would be high, disturbing the response by emergency institutions and consequently Disaster Governance. For example, in Puerto Saavedra, the emergency infrastructure including schools, fire stations, police departments, and the main area of commerce are below the inundation level (Figure 1). In contrast, rural and indigenous areas have less emergency infrastructure, yet the community headquarters and natural resources in these areas are located above the tsunami inundation zone. Thus, this could facilitate the community’s ability to adapt from tsunamis and, hence, Community Resilience. Additionally, in the indigenous area of M. Bajo, there are social headquarters, a school, water, forest and scrubland above the inundation area. Thus, where people could congregate obtain water, food, and firewood in the event of a tsunami. As such, in certain situations, rural and indigenous areas, versus urban areas, show better Disaster Governance and could be more likely to adapt from a tsunami.

Another aspect that influences our findings is that the distribution and allocation of resources during an emergency is not balanced. In the case studies, communal governments are the main bodies responsible for making decisions and making resources available to the community during a disaster. Specifically, the Emergency Operations Committee (COE) at the communal level makes most of the decisions during an emergency. The Municipality is part of the COE and mainly focuses its actions in urban areas through the Land Use Plan. As the Land Use Plan is only relevant to urban areas, the Disaster Governance of rural and indigenous areas is negatively impacted. As a result, rural and indigenous areas are disadvantaged compared to urban areas. Giving priority to intercommunal planning instruments, which have the capacity to regulate urban, rural and indigenous areas as a whole, could improve Disaster Governance in the study areas.

The political aspects of Disaster Governance [8,9,11,12] also influence our findings. A recent study has evaluated the physical and social causes of mortality resulting from a tsunami, finding that social aspects can be negatively affected by political decision taken in the territory [33]. The study highlights the fact that knowing the stock of social capital can influence mortality rates during a tsunami. In particular, mortality can be affected by the merging or creation of new localities. This information is relevant for the case studies because the Chilean coast is a highly dynamic environment where rural areas are often absorbed by urban zones. Indeed, in Mehuín and Puerto Saavedra future land use plans are proposed to include rural as well as urban areas. According to Aldrich and Sawada [33], the merging of rural and urban areas is a political condition that can diminish social capital because it causes people to lack identity with their new locality and representation in the political arena. This is why the authors recommend allocating funds to build the social ties among people and not only to recover physical infrastructure after a disaster. Such a recommendation can be of great value for improving Disaster Governance in the case studies presented here.

The findings of this study provide insight regarding the Disaster Management cycle, particularly with respect to the preparedness, response, and recovery stages. First, during the preparedness stage a more flexible evacuation plan should be developed for the case studies that includes all units of government (urban, rural, and indigenous). Particularly in the rural and indigenous areas, the emergency plan should diversify the emergency institutions involved during a disaster. This would assure the action of emergency institutions at lower governmental levels, which would provide efficient responses to all territorial scales. Training during the preparedness stage could be used to evaluate the equity in the quality of the response of existing emergency plans. Second, during the response stage, more fluidity in the assistance of institutions to all units of government could be achieved by diversifying the role of institutions. Specifically, members of institutions should be trained so that they can take on different roles in the event that other institutions fail. In this way, the redundancy of governance will be improved. Additionally, social institutions should be prepared in order to enhance social capital. This is particularly important in rural and indigenous areas where the action of emergency institutions is limited. Finally, during the recovery stage the allocation of funding and applications of grants should be adequately guided as to enhance Disaster Governance. Here we have found that Disaster Governance of urban, rural and indigenous areas differs. Hence, funding should be used to improve Disaster Governance in the areas and aspects that is particularly needed. This may be hard to achieve due to the political influence which has historically affected the distribution of funding [11,12]; however, it is necessary if Disaster Governance and hence, Community Resilience, are to be improved. In this sense, the division of the territory into urban, rural, and indigenous areas can hinder Governance. The increased population densities of urban areas greatly influence political will and thus the allocation of funding and the creation of laws. Regardless, the implementation of transitions zones in disaster prone areas can be rapidly implemented to improve disaster response. From this, physical and human infrastructure can be used to provide an equal, flexible and fluid response regardless of disparities in income, access to services, and political power.

Today, the relationship between government and society is not determined by the government alone. Disaster Governance is two-way where society and citizenship assume a higher level of responsibility and interaction. Disaster Governance is also political. The effectiveness of emergency actions for the adequate recovery and protection of communities depends on the bonds of society as well as on the state’s capacity and willingness to provide aid. This is particularly relevant in disaster scenarios where rapid as well as efficient responses are required to assure that communities are resilient: this can only be achieved by including all visions in a dynamic and flexible manner.

## Figures and Tables

**Table 1:** Roles of emergency institutions.

Governmental Institutions	Role
ONEMI	Plan, coordinate ^a^, and execute the activities that demand the operation of the National Civil Protection System in order to protect people, their assets, and the environment at national, regional, provincial and communal scales. Overall, the organization aims at prevention, mitigation, alertness, response and rehabilitation.
MINVU	Carry out the evaluation ^b^ (counting and description) of damaged houses in conjunction with local authorities. With the help of SERVIU, evaluates ^b^ the state of streets in the different cities of the region.
Municipality	Their actions are based on the Community Emergency Plan and consist of coordinating the evacuation ^c^ and subsequent delivery of supplies ^d^.
Police	Preliminary diagnosis of the effects of the disaster on citizenship. Ensure the maintenance of public order ^e^ throughout the region. Coordinate the provision of assistance to people and sectors after a catastrophe and collaborate with the evacuation ^c^.
Sea Municipal Authority	Collaborate with the evacuation ^c^ of the community and safeguard security ^e^. Provide emergency and evacuation information ^f^ in coordination with the communal COE. Conduct a post-tsunami emergency water search ^g^ plan.
Maritime Authority	Provide reports ^f^ on data from SHOA regarding possible tsunamis. Support transportation, search ^g^ for missing persons, aid in evacuation ^c^ and provide a registry ^b^ of coastal damage and the distribution of distressed stock ^d^, especially when water displacement is required.
Non-Governmental Organizations	Role
Hospital	Ensure continuity of medical care ^h^ in response to increased demand.
Health Post	Assist with the evacuation ^c^ of people with disabilities and older adults without family support. Perform medical care ^h^ in refuges.
CESCOF	Provide human and physical resources to maintain medical care ^h^.
Rescue Team	Support the search ^g^ and rescue activities with the corresponding agencies.
Firefighters	Evacuate ^c^ the population and establish safe zones.
Schools	Provide shelter ^i^ to the population during the emergency.
Electricity Company	Repair power supply ^j^. Work in coordination with the municipality to detect power outages.
Water Company	Keep ONEMI informed regarding the state of the water service ^k^ and provide an emergency team to address problems with the water supply.
Radio and Television	Deliver information ^f^ to the community in coordination with police and firefighters.

Sources: Regional Emergency Plan 2012, Los Ríos Region; Tsunami Action Plan, Mariquina Commune; Civil Protection Regional Plan 2004, La Araucanía Region. These were complemented with interviews in the field. Note on Table 1: The superscripts indicate the specific role. The following superscripts are associated with roles provided by multiple institutions: ^b^ (evaluation), ^c^ (evacuation); ^d^ (delivery of supplies and stock); ^e^ (security and public order); ^f^ (information and reports); ^g^ (search); ^h^ (medical care); and ^i^ (shelter). The following are associated with roles provided by one organization: ^a^ (coordination); ^j^ (power supply); and ^k^ (water services). ONEMI stands for the National Emergency Office. MINVU stands for the Ministry of Housing and Urbanism. CESCOF stands for Health and Family Health Center.

**Table 2:** Area of influence of the standard deviation ellipses.

Emergency Institutions	Mehuín		Queule		La Barra		Puerto Saavedra	
	Rotation	Area (km^2^)	Rotation	Area (km^2^)	Rotation	Area (km^2^)	Rotation	Area (km^2^)
ONEMI	99.47	27	6.66	2.91	-	-	15.07	2
MINVU	-	-	-	-	-	-	164.43	1
Municipality	21.71	3	9.79	217.28	33.72	74.03	68.71	74
Police	29.81	3	-	-	-	-	13.13	6
Sea Municipal Authority	31.8	3	51.12	0.23	-	-	-	-
Maritime Authority	-	-	-	-	-	-	14.46	4
Hospital	-	-	-	-	-	-	11.04	5
Health Post	-	-	62.94	2.77	24.26	41.97	-	-
CECOSF	105.28	9	-	-	-	-	-	-
Rescue Team	5.79	1	-	-	-	-	-	-
Firefighters *	174.77	1	13.97	4.37	44.21	11.72	1.94	4
Toltén Firefighters **	-	-	9.3	34.17	167.78	18.79	-	-
School	-	-	73.23	0.23	-	-	-	-
Electricity Company	-	-	68.48	1.11	-	-	24.31	1
Water Company	-	-	-	-	-	-	175.91	0.23
Radio and Television	-	-	-	-	-	-	135.91	29

Note: ** Toltén Firefighters also perform in the towns of La Barra and Queule. This is a communal firefighter department that collaborates with the local firefighter departments indicated in the table with an *.
